# Cortical Areas Associated With Mismatch Negativity: A Connectivity Study Using Propofol Anesthesia

**DOI:** 10.3389/fnhum.2018.00392

**Published:** 2018-10-02

**Authors:** Yun Zhang, Fei Yan, Liu Wang, Yubo Wang, Chunshu Wang, Qiang Wang, Liyu Huang

**Affiliations:** ^1^School of Life Science and Technology, Xidian University, Xi'an, China; ^2^Department of Anesthesiology, The First Affiliated Hospital of Xi'an Jiaotong University, Xi'an, China

**Keywords:** auditory mismatch negativity, EEG, propofol anesthesia, source analysis, wPLI

## Abstract

Auditory mismatch negativity (MMN) is an event-related potential (ERP) waveform induced by rare deviant stimuli that occur in a stream of regular auditory stimuli. The generators of MMN are believed to include several different cortical regions like the bilateral temporal and the right inferior frontal gyrus (IFG). However, exact cortical regions associated with MMN remain controversial. In this study, we compared the number of long-distance connections induced by the standard and deviant stimuli during awake state and propofol anesthesia state to identify the cortical areas associated with the generation of MMN. In awake state, we find that deviant stimuli synchronize more information between the right frontal and temporal than standard stimuli. Moreover, we find that the deviant stimuli in awake state activate the bilateral frontal, central areas, the left temporal and parietal areas as compared to the anesthesia state, whereas the standard stimuli do not. These results suggest that, in addition to the bilateral temporal and the right IFG, the bilateral frontal and centro-parietal regions also contribute to the generation of MMN.

## Introduction

Auditory MMN is an ERP waveform induced by rare deviant stimuli that occur in a stream of regular auditory stimuli (Näätänen et al., 2001; Garrido et al., 2009a; Paavilainen, 2013). These deviant stimuli can be obtained by changing the frequency, intensity, duration, or other auditory characteristics of the standard stimuli. The peak of MMN usually occurs at 100–200 ms after stimuli with maximal amplitudes over fronto-central scalp regions.

Currently, the predictive coding is the leading hypothesis for explaining the neural mechanism that underlies the generation of MMN (Garrido et al., 2009b). It posits that the generation of MMN involves a hierarchical neural network interconnected with recurrent connections (Schmidt et al., 2013). The low-level brain regions are responsible for the abstraction of the auditory input, whereas the high-level brain regions are responsible for predicting the upcoming stimulus (Friston, 2005). MMN is generated when an input violates the top-down prediction. Moreover, the repeated standard stimuli which reduces the strength of bottom-up connection could also contribute to the observed MMN waveform (Garrido et al., 2009b).

The dynamic casual modeling (DCM) is the primary tool to test the predictive coding hypothesis (Garrido et al., 2008). In DCM, a model with pre-selected brain regions is constructed to fit the observed MMN waveform obtained from neuroimaging data (Garrido et al., 2009a). An accurate DCM model rests on the properly assigned cortical generators of MMN (Friston, 2003). Most of studies using DCM selected the bilateral superior temporal gyrus and the right inferior gyrus to fit the measured MMN waveform (Garrido et al., 2008, 2009a; Cooray et al., 2016; Ranlund et al., 2016). In addition, experimental evidences suggest that the auditory cortex and the frontal cortex are both activated during MMN (Rinne et al., 2000; Opitz et al., 2002; Doeller et al., 2003). The involvement of bilateral superior temporal gyrus has been increasingly supported by experimental evidences, however, the role of the frontal cortex generators remains unclear (Alho, 1995; Opitz et al., 2002; Doeller et al., 2003; Rosburg, 2003; Molholm et al., 2005; Rosburg et al., 2005). Therefore, it is of great significance to locate the exact generators of MMN, which may lead to an accurate DCM model.

A typical MMN waveform composes of a temporal component and a frontal component (Baldeweg, 2007). The temporal component of MMN is originated from the auditory cortex and it represents the auditory feature extraction process, whereas the frontal component may be caused by the high level cognitive function such as attention switch (Joos et al., 2014). It has been shown that as compared to standard stimuli, the deviant stimuli induced more brain activation in both temporal lobe and frontal lobe (Molholm et al., 2005). Hence, we can infer that the deviant stimuli are considered as more cognitively demanding as compared to the standard stimuli.

The workspace theory predicts that cognitive demanding tasks will result in an increased information integration among discrete cortical regions (Dehaene and Naccache, 2001; Baars, 2002). Therefore, according to the workspace theory, the cortical areas that are responsible for the generation of MMN can be identified by comparing the cortical regions activated by standard and deviant stimuli respectively. One of the early attempts found that deviant stimuli induce increased long-distance information synchronization among discrete cortex regions as compared to standard stimuli (Nicol et al., 2012). However, they only analyzed data on sensor level which prohibited them to pinpoint the cortical regions that are responsible for the generation of MMN.

An alternative approach to identify the cortical associated with the generation of MMN is to abolish the high-level cognitive function that is required for generating MMN during the presentation of deviant stimuli. Various anesthetic agents can achieve this target by blocking the information transmission across multiple brain regions (Lee et al., 2009, 2013; Ku et al., 2011). It has been shown that the primary auditory perception of the brain is still active under the deep sedation (Ku et al., 2011; Lee et al., 2013), whereas the auditory stimulation processing is totally broken down at deep level of anesthesia (Heinke et al., 2004; Koelsch et al., 2006).

In the present study, we hypothesized that, based on the workspace theory, deviant stimuli would induce increased long-distance connectivity across distinct brain regions as compared to the standard stimuli, and these connections should be blocked by propofol induced anesthesia. To test this hypothesis, we recorded EEG signal during an MMN paradigm under awake and propofol-induced anesthesia states, respectively. Then, source analysis was conducted to identify the cortical regions with different long/short-distance connections between the brain networks involved in processing the two kinds of stimuli under both awake and propofol-induced anesthesia states. Our findings might provide further evidence on the cortical regions associated with MMN and shed lights on the generation mechanism of MMN.

## Materials and methods

### Subjects

This study was approved by First Affiliated Hospital of Xi'an Jiaotong University and complied with the guidelines of the Declaration of Helsinki. Written informed consent was obtained from all participants. Totally, 25 healthy subjects (age = 35.5 ± 5.3) participated in this study. All the subjects are drug-free and allergy-free to propofol. Exclusion criteria included a history of neurological, psychiatric or hearing diseases. Each participant was asked to fast for at least 8 h before propofol administration.

### Data acquisition

Scalp EEG data was acquired from 64 Ag/AgCl electrodes which were placed according to the International 10–10 system. The reference electrodes were placed at bilateral mastoids. Two electrodes were placed above and below the left eye to record the vertical electro-oculogram (VEO). The impedance of all electrodes was kept below 5 kΩ. The EEG data was recorded using SynAmps amplifier (Neuroscan Inc.,) with a sampling rate of 1,000 Hz.

### Stimuli

The MMN paradigm contained three kinds of pure sinusoidal tones with different pitch: a standard tone (500 Hz, *n* = 200) and two deviant tones (450 Hz, *n* = 50; 550 Hz, *n* = 50). There are 300 randomly ordered tones in each block. The duration of tones was 75 ms with a 10 ms rising/falling time, and the inter-stimulus interval between each stimulus was 1000 ms. E-prime software (Pittsburg, PA, USA) was used to design and present these pure tones (80 dB) through a pair of earphones (Sony, MDR EX450).

### Anesthesia infusion

All the experiments were performed by two experienced anesthesiologists. Propofol was employed as the sole anesthetic in the experiment and it was administered by a target-controlled infusion device (TCI, Injectomat TIVA Agilia). During the experiment, vital signs including heart rate, blood pressure, respiratory rate and pulse oximetry (SpO_2_) were continuously monitored by using intra-operative monitors (MP50, PHILIPS). The anesthesia depth was tracked by using the bispectral index monitor. The bispectral index (BIS) is a processed EEG parameter and it has been widely adopted in the clinical settings for assessing the level of anesthesia during surgery (Myles et al., 2004). The BIS value ranges from 0 (flat line EEG) to 100 (fully awake). In the present study, the awake state was defined as the BIS value is equal or >90, whereas the deep anesthesia state is defined as the BIS within the range of 35–45.

### Procedure

Subjects comfortably laid on the bed after EEG cap and headphone were set. They were asked to close their eyes and stay relaxed during the experiment. The experiment was carried out following the procedures as below:

1. Awake state: In this state, subjects were asked to stay awake with eye closed. 2 min resting EEG data and 4 min ERP data were collected.
2. Anesthesia: The propofol plasma target concentration was first set at 1–2 μg/ml. A laryngeal was placed after subjects lost their consciousness and connected to a ventilator, which was set to the SIMV mode. The tidal volume was set at 8–10 ml/kg. The respiratory rate was 12 breaths/minute. The end-tidal carbon dioxide partial pressure was maintained at 35–45 mmHg and the plasma propofol target concentration was set at 4–4.5 μg/ml. 2 min resting EEG data and 4 min ERP data were collected while the BIS value was maintained around 40.
3. Recovery of anesthesia: The reverse protocol was initiated by setting the plasma concentration of propofol to 0 μg/ml. Then, the subjects were pushed into the anesthesia recovery room to wait for the complete recovery.

In this study, we analyzed the 4 min ERP data from each state, the resting data were kept for other studies.

### EEG preprocessing and ERP analysis

**Figure 1:**
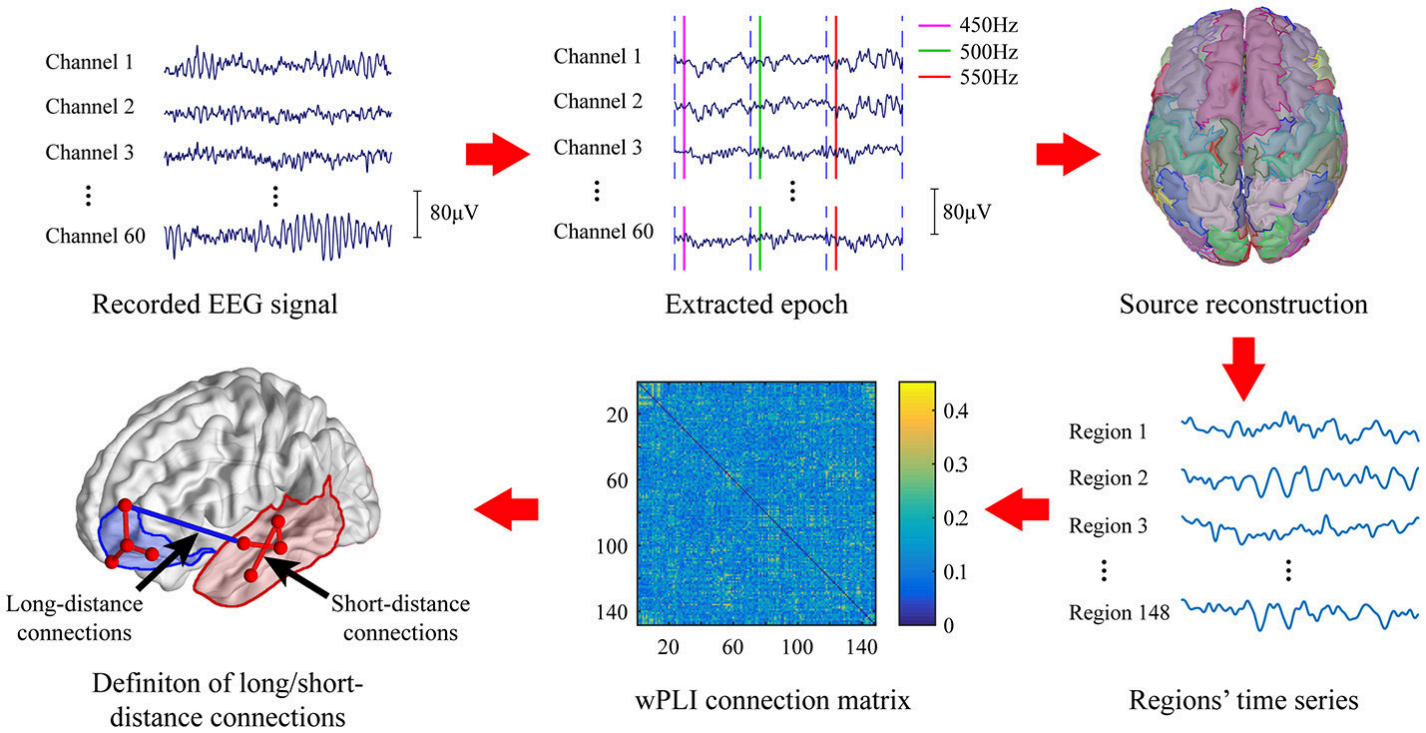
EEG data analysis pipeline.

The EEG signal processing pipeline was given in Figure 1. Raw EEG signal was first band-pass filtered at 0.5–40 Hz to remove baseline drift and power line noise. The segments of data contain excessive fluctuation and eye movement were manually removed.

Epochs of standard stimuli (500 Hz) and deviant stimuli (450 Hz; 550 Hz) were extracted with 100 ms before to 700 ms after the stimulus was given. The clean epochs were baseline-corrected by subtracting the mean amplitude of the first 100 ms of each epoch. Then, the extracted epochs were grouped and averaged according to the types of stimuli, i.e., the standard stimuli or deviant stimuli. Moreover, we only considered the deviant stimuli which is preceded by at least 3 continuous standard tone. The MMN waveform was obtained by subtracting the averaged EEG signal of standard stimuli from that obtained on deviant stimuli. Subjects with no MMN waveform were excluded from further analysis.

### Source reconstruction

Since long-range synchronization among cortical regions was mostly presented in the high frequency band (Nicol et al., 2012), only gamma band was considered in the present study. The clean epochs of EEG signal were first band-pass filtered in the frequency band of gamma band (30–40 Hz). Then, source reconstruction was performed to localize cortical activation by using Brainstorm (Tadel et al., 2011). The MNI model (Colin27) (Collins et al., 1998) was used as the standard head model and the standard Neuroscan Quik-Cap64 registration channel file was used as the electrode registration. The forward model was first calculated using the OpenMEEG boundary element method (Gramfort et al., 2010). We selected the weighted minimum norm estimator (wMNE) (Baillet et al., 2001) to estimate the activation of 15002 dipoles distributed over the entire cortex. Then, these dipoles were divided into 148 regions of interest (ROI) based on the Destrieux atlas (Destrieux et al., 2010). Activity of each ROI was formed by averaging the current source density of all voxels within that region. These 148 ROIs were further divided into 14 areas according to their anatomical location on the cortex including bilateral prefrontal(LPF/RPF), bilateral frontal(LF/RF), bilateral central(LC/FC), bilateral temporal(LT/RT), bilateral occipital(LO/RO), and bilateral cingulum(LCing/RCing).

### Network analysis

Recent studies have shown that the data segment around the maximum amplitude of ERP or MMN is suitable for studying the brain functional network related to its generation (Nicol et al., 2012; Karamzadeh et al., 2013). Hence, we selected the data segment according to the maximum amplitude of MMN that is identified for each subject at the awaken state for constructing the brain functional network. Furthermore, it has been shown that the anesthetics can attenuate the amplitude of ERP, whereas the latency of the ERP is left unchanged (Ozgoren et al., 2010). Therefore, we extracted EEG data in the anesthetic state from the same time window as identified in the awaken state for estimating the brain functional connectivity in the absence of observable MMN waveform. In doing so, the difference in functional connectivity at the same time frame relative to the onset of stimuli between awaken and anesthetic state would reflect the changes that is related to the generation of MMN.

**Figure 2:**
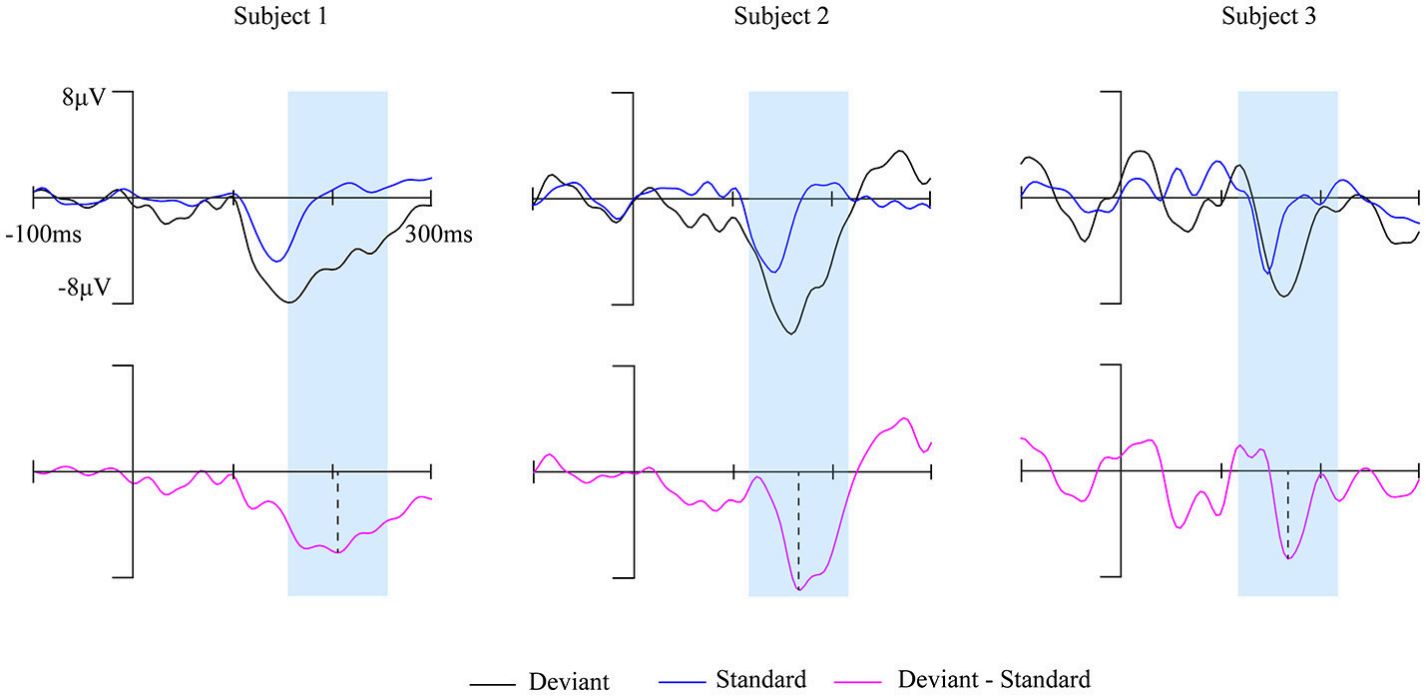
The MMN waves of three subjects at Fz electrode during awake state. The time window of 100 ms centered on the peak of each subject's MMN responses (magenta solid line) was marked by the blue shaded area.

The brain activity in a 100 ms temporal window which is centered at the peak of MMN waveform was extracted (marked blue in Figure 2) to construct the brain network. The 148 ROIs were defined as nodes and the connectivity strength between the nodes were defined as edges.

The weighted phase lag index (wPLI) was employed to measure the connectivity between a pair of ROIs. The reason of selecting wPLI is due to its robustness against the influence of volume conduction (Vinck et al., 2011; Lau et al., 2012). The wPLI was defined as:

(1) $$\begin{array}{r} {\text{wPLI}}_{ij}=\frac{1}{n}\overset{n}{\underset{t=1}{\sum }}|\langle \frac{\left|\sin\left(S_{ijt}\right)\right|}{\sin\left(S_{ijt}\right)}\rangle | \end{array}$$

where *n* is the number of trials, *t* is the index of trial, *S*_*ijt*_ represents a vector of phase differences between region *i* and region *j* at each time point within the *t*-th trial. The phase of brain activity of each ROI was computed from the signal x(t) and its corresponding Hilbert transform $\tilde{x}$(t).

(2) $$\begin{array}{r} S_{t}={\text{tan}}^{-1}\frac{\tilde{x}\left(t\right)}{x\left(t\right)} \end{array}$$

The obtained wPLI ranges from 0 to 1 in which 1 indicates signal *i* always leads or lags signal *j* in all trials and it equals to 0 indicates that the phase difference between two signals are completely random across trials.

To prevent false connections, the obtained wPLI of all possible pairs were pruned by preserving the strongest connections above a threshold. Previous research showed that the average degree of networks must be larger than 2 × ln *N* to maintain the integrity of network, where *N* is the number of nodes in the network (Watts and Strogatz, 1998). In the present study, the corresponding lowest threshold is about 2 × ln 148 = 6%. Hence, we constructed networks with a wide range of connection densities ranging from 6 to 90%.

### Long/short-distance connections

In source analysis, we grouped the 148 ROIs into 14 cortical areas according to its anatomic location. Similar to the previous work (Kitzbichler et al., 2011; Nicol et al., 2012), we then defined the functional connections among these cortical areas as the long-distance connections, and the connections within each cortical area as the short-distance connections.

The weighted value of long/short connections was calculated as the sum of the corresponding wPLI values in the connection matrix. The long-distance connection of the whole brain network was given as

(3) $$\begin{array}{r} L_{all}=\overset{14}{\underset{k=1}{\sum }}L_{k} \end{array}$$

The short-distance connection of the whole brain network was defined as:

(4) $$\begin{array}{r} S_{all}=\overset{14}{\underset{k=1}{\sum }}S_{k} \end{array}$$

where *k*∈[1, 2, 3, …, 14] is the index of the 14 cortical areas, *L*_*k*_ and *S*_*k*_ represent the connection strength of long/short-distance connects, respectively. The ratio between the value of the long-distance connections and the short-distance connections was defined as

(5) $$\begin{array}{r} Ratio=\frac{L_{all}}{S_{all}}=\frac{\sum_{k=1}^{14}L_{k}}{\sum_{k=1}^{14}S_{k}} \end{array}$$

We first compared the *Ratio* of the brain networks involved in processing the two kinds of stimuli in awake state. If there was a statistically significant difference on *Ratio*, then we compared *L*_*all*_ and *S*_*all*_ obtained from two kinds of stimuli to find out the cause of the difference in *Ratio*. Then, we compared the obtained measures between awake and anesthesia states.

Statistical comparison of the number of long/short-distance connections and *Ratio* between the two states and between the two kinds of stimuli were conducted by using paired *t*-test. *P* < 0.05 was considered as statistically significant.

## Results

### MMN

**Figure 3:**
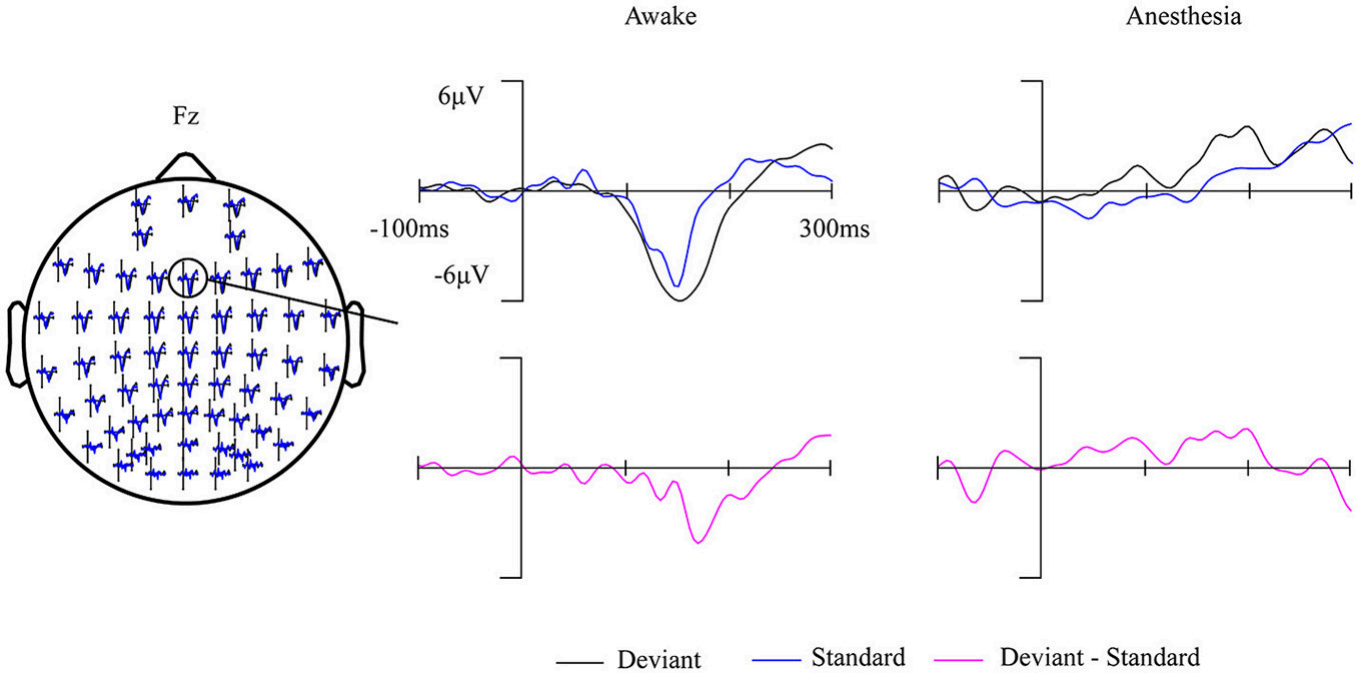
Grand-average (14 subjects) ERP waves in awake state and in anesthesia overlaid on a scalp-map of 60 EEG electrodes with the ERP response at Fz were enlarged.

Fourteen out of 25 subjects showed a typical MMN waveform during awake state, the other 11 subjects with no clear MMN waveform were excluded from further analysis. The obtained MMN waveform from three representative subjects at Fz electrode during awake state was provided in Figure 2. It can be observed that both standard stimuli and deviant stimuli induced a negative going response after the stimuli. The MMN is the negative differential response between deviant stimuli and standard stimuli at the Fz electrode. As shown in Figure 3, the observed MMN wave vanished at anesthesia state.

### Long/short-distance connections

**Figure 4:**
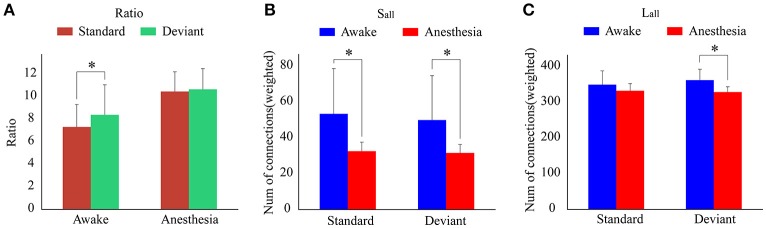
**(A)** The *Ratio* of the brain network for standard stimuli and deviant stimuli before and after anesthesia. **(B)** The *S*_*all*_ and **(C)** the *L*_*all*_ of the network during awake state and anesthesia for the standard stimuli and deviant stimuli. (*P < 0.05).

As shown in Figure 4A and Table 1, in awake state, the *Ratio* between the long/short-distance connections induced by deviant stimuli was significantly higher than that induced by standard stimuli. However, no difference was found during anesthesia state. It was noticed that the *Ratio* of two kinds of stimuli were both higher during anesthesia than during awake state. We then compared the difference of the *S*_*all*_ and the *L*_*all*_ of two kinds of stimuli during awake state and anesthesia to investigate the cause of the increase in *Ratio*. As shown in Figures 4B,C and Table 2, both *S*_*all*_ and *L*_*all*_ were decreased under anesthesia. However, the reduction on *S*_*all*_ was significantly higher than *L*_*all*_, which results in an increase in *Ratio* during anesthesia state.

**Table 1:** The Ratio of the brain network for standard stimuli and deviant stimuli before and after anesthesia.

**State**	**Standard**		**Deviant**			
	**Mean**	**Std**	**Mean**	**Std**	***t***	***p***
Awake	7.310012	1.995199	8.385939	2.664839	−2.1662	0.0495
Anesthesia	10.45203	1.751373	10.65118	1.841951	−0.348	0.7334

**Table 2:** The *S*_*all*_ and *L*_*all*_ of the network in awake state and anesthesia for two kinds of stimuli.

***S*_**all**_**	**Awake**		**Anesthesia**			
	**Mean**	**Std**	**Mean**	**Std**	***t***	***p***
Standard	53.44007	25.25337	32.57088	5.087076	2.946319	0.011352
Deviant	49.95545	24.77402	31.61348	4.708451	2.916409	0.012023
***L***_*all*_						
Standard	349.5844	38.81111	332.7096	19.90393	1.448288	0.171224
Deviant	362.1961	30.58026	328.9456	14.94464	3.787093	0.002262

**Figure 5:**
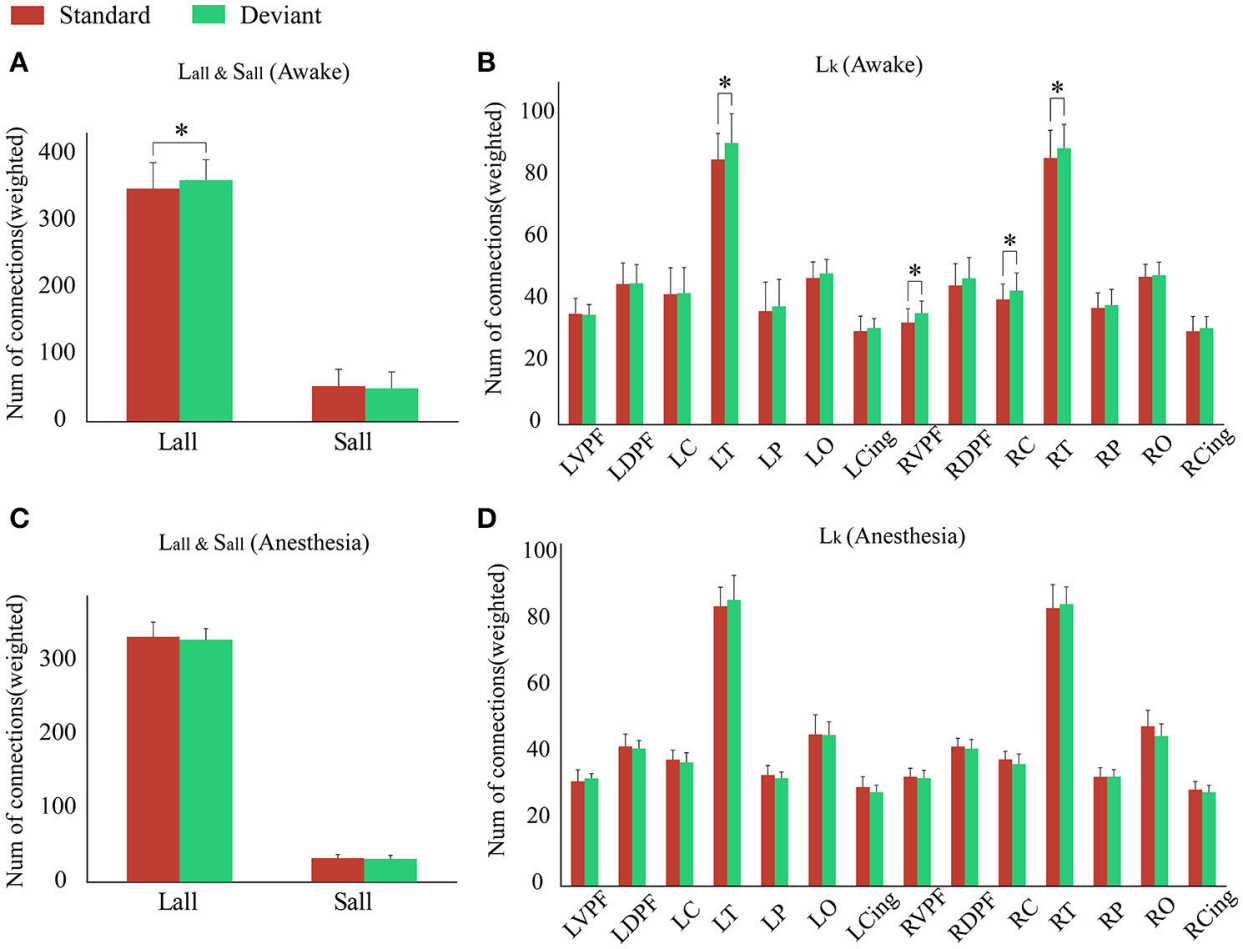
During **(A)** awake state and **(C)** anesthesia state, the *L*_*all*_ and the *S*_*all*_ of the network for the standard stimuli and deviant stimuli. During **(B)** awake state and **(D)** anesthesia state, the number of *L*_*k*_ of each cortical region for standard stimuli and deviant stimuli. (*P < 0.05).

Since the changes in *Ratio* between the standard and deviant stimuli might be caused by the changes of the *L*_*all*_ or the *S*_*all*_, we compared the *L*_*all*_ and the *S*_*all*_ of the two kinds of stimuli in both awake state and anesthesia state. We found that in awake state, the *L*_*all*_ of deviant stimuli was significantly higher than the standard stimuli, while the *S*_*all*_ of these two kinds of stimuli had no significantly difference (Figure 5A and Table 3). This finding suggested that increased Ratio induced by deviant stimuli was mainly caused by the increase of long-distance connections. Furthermore, we compared the differences in the number of long-distance connections of each cortical area to identify which region contributed the most to the increased *L*_*all*_ of deviant stimuli. As shown in Figure 5B and Table 4, deviant stimuli induced more long-distance connections in the right prefrontal, central and bilateral temporal areas as compared to the standard stimuli. On the other hand, there were no statistically significant differences in *L*_*all*_, *S*_*all*_, and *L*_*k*_ at each cortical region between the standard and deviant stimuli during anesthesia state (Figures 5C,D, Tables 3, 4).

**Table 3:** In awake state and anesthesia state, the *L*_*all*_ and the *S*_*all*_ of the network for two kinds of stimuli.

**Awake state**	**Standard**		**Deviant**			
	**Mean**	**Std**	**Mean**	**Std**	***t***	***p***
**L**_**all**_	349.5844	38.81111	362.1961	30.58026	−1.862	0.0854
**S**_**all**_	53.44007	25.25337	49.95545	24.77402	0.573408	0.576154
**ANESTHESIA STATE**						
**L**_**all**_	332.7096	19.90393	328.9456	14.94464	0.5994	0.5592
**S**_**all**_	32.57088	5.087076	31.61348	4.708451	0.9657	0.3518

**Table 4:** During awake and anesthesia state, the number of long-distance connections of each cortical region for standard stimuli and deviant stimuli.

**Awake state**	**Standard**		**Deviant**		***t***	***p***
	**Mean**	**Std**	**Mean**	**Std**		
LVPF	35.84441	4.993638	35.45339	3.350978	0.383865	0.707283
LDPF	45.41966	6.825106	45.66346	6.095389	−0.17311	0.865228
LC	42.16068	8.527032	42.51223	8.257726	−0.28584	0.779505
LT	85.73301	8.383562	91.03238	9.494782	−2.52204	0.025505
LP	36.67181	9.40771	38.22	8.762029	−1.21905	0.244484
LO	47.35155	5.270706	48.85374	4.549244	−0.98445	0.342861
LCing	30.26029	4.788322	31.24875	3.042433	−0.92269	0.372977
RVPF	33.00135	4.418252	36.02398	3.916595	−2.99545	0.010329
RDPF	45.0071	7.014148	47.27165	6.764838	−1.66331	0.120157
RC	40.47954	5.003315	43.28452	5.736258	−2.17189	0.048957
RT	86.14592	9.022009	89.36555	7.71195	−2.35434	0.034942
RP	37.74266	4.858752	38.67313	5.066942	−0.91802	0.375329
RO	47.81438	4.011608	48.30463	4.210533	−0.34947	0.732331
RCing	30.164	4.799899	31.19539	3.76657	−1.39089	0.187609
**ANESTHESIA STATE**						
LVPF	31.64545	3.489466	32.52888	1.429545	−0.93702	0.365829
LDPF	42.20827	3.710367	41.52693	2.42279	0.535166	0.601576
LC	38.20362	2.849107	37.33589	2.918319	0.774336	0.452586
LT	84.44878	5.752601	86.37667	7.411751	−0.77706	0.451035
LP	33.53955	2.874027	32.6164	1.855945	1.01135	0.330303
LO	45.80071	5.945685	45.62789	4.012679	0.082833	0.935246
LCing	29.922	3.10444	28.37361	2.119977	1.643734	0.124185
RVPF	33.01758	2.588315	32.56957	2.413068	0.507748	0.620135
RDPF	42.13584	2.492122	41.49173	2.764988	0.64699	0.528897
RC	38.24165	2.439038	36.82709	3.024949	1.228173	0.241155
RT	83.8783	7.072592	85.09531	5.206525	−0.7026	0.494695
RP	32.99575	2.758199	33.0496	2.1234	−0.05706	0.955365
RO	48.26436	4.829606	45.28658	3.682514	1.784733	0.097646
RCing	29.13361	2.458428	28.34803	2.131624	0.864169	0.403159

**Figure 6:**
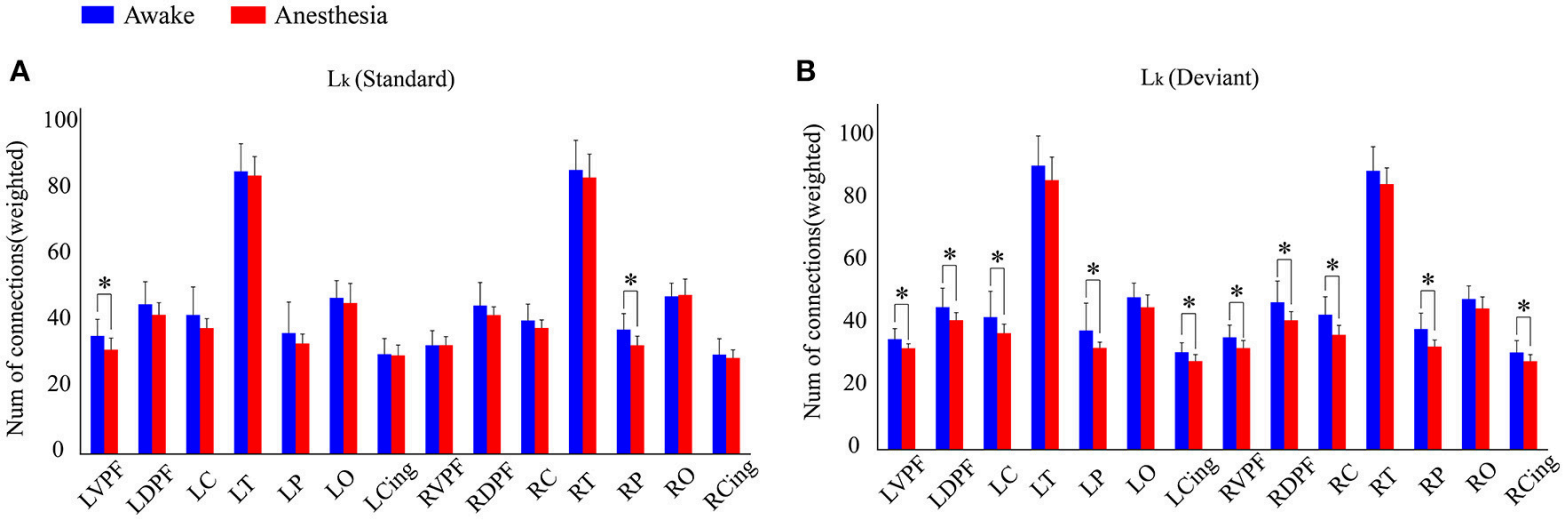
The number of *L*_*k*_ of each cortical region **(A)** in awake state and **(B)** anesthesia state for standard stimuli and deviant stimuli (*P < 0.05).

We also compared the number of long-distance connections induced by standard stimuli and deviant stimuli between the awake and anesthesia states. As compared to the state of anesthesia, standard stimuli in the awake state induced greater number of long-distance connections at left prefrontal and right central areas (Figure 6A and Table 5). In contrast, the number of long-distance connections induced by deviant stimuli were higher at the bilateral frontal, central, parietal and cingulate areas in awake state (Figure 6B and Table 5).

**Table 5:** The number of long-distance connections of each cortical region in awake state and anesthesia for standard and deviant stimuli.

**Standard**	**Awake**		**Anesthesia**		***t***	***p***
	**Mean**	**Std**	**Mean**	**Std**		
LVPF	35.84441	4.993638	31.64545	3.489466	2.313753	0.037684
LDPF	45.41966	6.825106	42.20827	3.710367	1.80647	0.094038
LC	42.16068	8.527032	38.20362	2.849107	1.864204	0.085028
LT	85.73301	8.383562	84.44878	5.752601	0.492529	0.630559
LP	36.67181	9.40771	33.53955	2.874027	1.190344	0.255195
LO	47.35155	5.270706	45.80071	5.945685	0.650261	0.526849
LCing	30.26029	4.788322	29.922	3.10444	0.206607	0.839518
RVPF	33.00135	4.418252	33.01758	2.588315	−0.01118	0.991247
RDPF	45.0071	7.014148	42.13584	2.492122	1.515237	0.153644
RC	40.47954	5.003315	38.24165	2.439038	1.331514	0.205898
RT	86.14592	9.022009	83.8783	7.072592	0.712293	0.488871
RP	37.74266	4.858752	32.99575	2.758199	2.77834	0.015666
RO	47.81438	4.011608	48.26436	4.829606	−0.24341	0.811488
RCing	30.164	4.799899	29.13361	2.458428	0.645172	0.530038
**DEVIANT**						
LVPF	35.45339	3.350978	32.52888	1.429545	3.576705	0.003379
LDPF	45.66346	6.095389	41.52693	2.42279	2.321895	0.037118
LC	42.51223	8.257726	37.33589	2.918319	2.228012	0.044162
LT	91.03238	9.494782	86.37667	7.411751	1.59056	0.135723
LP	38.22	8.762029	32.6164	1.855945	2.374266	0.033667
LO	48.85374	4.549244	45.62789	4.012679	1.938141	0.074632
LCing	31.24875	3.042433	28.37361	2.119977	3.113923	0.008223
RVPF	36.02398	3.916595	32.56957	2.413068	2.441846	0.029664
RDPF	47.27165	6.764838	41.49173	2.764988	2.542942	0.024518
RC	43.28452	5.736258	36.82709	3.024949	4.0913	0.001274
RT	89.36555	7.71195	85.09531	5.206525	1.748547	0.103927
RP	38.67313	5.066942	33.0496	2.1234	3.805544	0.002184
RO	48.30463	4.210533	45.28658	3.682514	1.689686	0.114912
RCing	31.19539	3.76657	28.34803	2.131624	2.268673	0.040969

## Discussion

In this study, we compared the number of long-distance connections induced by the standard and deviant stimuli during awake state and propofol induced anesthesia state to identify the cortical areas which are associated with the generation of MMN.

We found that in the awake state, deviant stimuli induced a greater number of long-distance connections than standard stimuli due to its high demand on cognitive function. This result was in consistent with the hypothesis of the global workspace theory (Kitzbichler et al., 2011; Nicol et al., 2012). The increment of long-distance connections was mainly distributed over the right prefrontal, bilateral temporal areas, which is in consistent with the previous studies (Rinne et al., 2000; Opitz et al., 2002; Doeller et al., 2003). Additionally, we found that the right central area also contributed to the increment of long-distance connections induced by deviant stimuli.

By comparing the measurements of brain networks involved in processing the two kinds of stimuli between awake and anesthesia state, we found that deviant stimuli induced a greater number of long-distance connections at the bilateral prefrontal, central, parietal and cingulate areas in awake state than that in anesthesia state. However, significant difference between two states induced by standard stimuli only existed on two cortical regions: left prefrontal and right parietal areas. As shown in Figure 5C, no differences existed between the brain network responsible for processing the standard stimuli and deviant stimuli during anesthesia. Hence, we could infer that during anesthesia, the brain networks involved in processing the two kinds of stimulus were suppressed to similar level. Furthermore, there were no significant difference in the number of long-distance connections induced by standard stimuli between the awake state and anesthesia state, while the number of long-distance connections induced by deviant stimuli was significantly suppressed during anesthesia state as compared to the awake state (Figures 4B,C). Hence, the cortical regions which may contributed to the differences of the long-distance connections induced by the deviant stimuli between the awake state and anesthesia state can be considered as the cortical regions associated with the generation of MMN.

Our results revealed that in the awake state, deviant stimuli induced more long-distance connections at bilateral temporal regions. However, the number of long-distance connections induced by deviant stimuli had no significant difference between the two states at bilateral temporal regions. There were several studies suggested that the cortical regions that are responsible for higher-level cognitive process is more sensitive to anesthesia as compared to the lower-level sensory cortex. The temporal cortex is responsible for the perceptual function of the auditory signal, which belongs to the low-level cortex and it is less sensitive to anesthesia, hence the number reduction on long-distance connections at temporal cortex during anesthesia is not as obvious as observed in other cortical areas.

The temporal cortex is responsible for forming sensory memory model, while the frontal cortex is responsible for comparing the memory model to the input stimuli. The sensory memory model will be adjusted when the frontal cortex finds that the current stimulus and the model does not match, and this process would result in the MMN (Garrido et al., 2009b). Our results suggested that the frontal regions were closely related to the generation of MMN, which is in line with the prediction of the predictive coding theory. In other words, the generation of MMN relies on the communication between the frontal and temporal cortex.

Several studies have suggested that the involvement of frontal lobe might be related to a specific MMN paradigm (Näätänen and Alho, 1997; Shestakova et al., 2002; Molholm et al., 2005). The right IFG was activated in the frequency condition, whereas the activation of the left IFG was observed in the duration condition. Most recent researches have selected bilateral temporal and right frontal cortex as the prior model to analyze the MMN response to pitch by using DCM (Garrido et al., 2008; Cooray et al., 2016; Ranlund et al., 2016). In this study, we found that the MMN to pitch did not only activate the right IFG, but also activate the left prefrontal. This finding is in line with previous studies (Lin et al., 2007; Wehner et al., 2007; Hsiao et al., 2010; Cheng et al., 2013).

Our results also suggested the parietal region (Kasai et al., 1999; Schall et al., 2003; Marco-Pallarés et al., 2005; Molholm et al., 2005) and the central region were associated with the generation of MMN (Molholm et al., 2005; Novitski et al., 2006; Fulham et al., 2014). The involvement of the parietal cortex might be supported by the anatomical connection from the parietal and prefrontal to the temporal cortex (Makris et al., 2005; Schmahmann et al., 2008; Kamali et al., 2014). Several fMRI and MEG studies have found that the fronto-temporo-parietal network might play a role in generating MMN (Schall et al., 2003; Cheng et al., 2013). Our results suggested that the generation of MMN activated a wide fronto-temporo-parietal network.

There are several limitations of our study. We used the standard Colin27 head model as the standard head model of every participant in source analysis, which might slightly bias the accuracy of the source reconstruction. Moreover, the EEG signal only record the neural activities on the scalp, the activation of the inner cerebral hemisphere, such as cingulate gyrus, cannot be accurately observed even by using the source analysis. Hence, we excluded the involvement of cingulate gyrus during the generation of MMN.

In conclusion, by comparing the brain networks involved in processing the standard and deviant stimuli in the MMN paradigm during awake and anesthesia state, we observed the increased number of long-distance connections originate from the bilateral prefrontal, temporal, central and parietal cortices, which suggested that the generation of MMN would require communications among prefrontal, temporal, and centro-parietal regions.

## Author contributions

YZ processed the data and wrote the manuscript. FY recruited subjects, performed data collection, and revised the manuscript. LW performed the data collection and processing and wrote the manuscript. YW performed data processing and revised the manuscript. CW performed the experiments. QW was responsible for designing the study and manuscript editing. LH designed the study and reviewed the manuscript. All authors contributed to the completion of the manuscript.

### Conflict of interest statement

The authors declare that the research was conducted in the absence of any commercial or financial relationships that could be construed as a potential conflict of interest. The reviewer DO and handling Editor declared their shared affiliation.

## References

[B1] Alho K. (1995). Cerebral generators of mismatch negativity (MMN) and its magnetic counterpart (MMNm) elicited by sound changes. Ear Hear. 16, 38–51. 10.1097/00003446-199502000-00004 7774768

[B2] Baars B. J. (2002). The conscious access hypothesis: origins and recent evidence. Trends Cogn. Sci. 6, 47–52. 10.1016/S1364-6613(00)01819-2 11849615

[B3] Baillet S. Mosber J. C. Leaby R. M. (2001). Electromagnetic Brain Mapping. IEEE Signal Process. Mag. 18, 14–30. 10.1109/79.962275

[B4] Baldeweg T. (2007). ERP repetition effects and mismatch negativity generation: a predictive coding perspective. J. Psychophysiol. 21, 204–213. 10.1027/0269-8803.21.34.204

[B5] Cheng C. H. Baillet S. Hsiao F. J. Lin Y. Y. (2013). Effects of aging on neuromagnetic mismatch responses to pitch changes. Neurosci. Lett. 544, 20–24. 10.1016/j.neulet.2013.02.063 23562510

[B6] Collins D. L. Zijdenbos A. P. Kollokian V. Sled J. G. Kabani N. J. Holmes C. J.. (1998). Design and construction of a realistic digital brain phantom. IEEE Trans. Med. Imaging 17, 463–468. 10.1109/42.712135 9735909

[B7] Cooray G. K. Garrido M. I. Brismar T. Hyllienmark L. (2016). The maturation of mismatch negativity networks in normal adolescence. Clin. Neurophysiol. 127, 520–529. 10.1016/j.clinph.2015.06.026 26189210

[B8] Dehaene S. Naccache L. (2001). Towards a cognitive neuroscience of consciousness: basic evidence and a workspace framework. Cognition 79, 1–37. 10.1016/S0010-0277(00)00123-2 11164022

[B9] Destrieux C. Fischl B. Dale A. Halgren E. (2010). Automatic parcellation of human cortical gyri and sulci using standard anatomical nomenclature. Neuroimage 53, 1–15. 10.1016/j.neuroimage.2010.06.010 20547229 PMC2937159

[B10] Doeller C. F. Opitz B. Mecklinger A. Krick C. Reith W. Schröger E. (2003). Prefrontal cortex involvement in preattentive auditory deviance detection: neuroimaging and electrophysiological evidence. Neuroimage 20, 1270–1282. 10.1016/S1053-8119(03)00389-6 14568496

[B11] Friston K. (2003). Learning and inference in the brain. Neural Netw. 16, 1325–1352. 10.1016/j.neunet.2003.06.005 14622888

[B12] Friston K. (2005). A theory of cortical responses. Philos. Trans. R. Soc. B Biol. Sci. 360, 815–836. 10.1098/rstb.2005.1622 15937014 PMC1569488

[B13] Fulham W. R. Michie P. T. Ward P. B. Rasser P. E. Todd J. Johnston P. J.. (2014). Mismatch negativity in recent-onset and chronic schizophrenia: a current source density analysis. PLoS ONE 9:e100221. 10.1371/journal.pone.0100221 24949859 PMC4064992

[B14] Garrido M. I. Friston K. J. Kiebel S. J. Stephan K. E. Baldeweg T. Kilner J. M. (2008). The functional anatomy of the MMN: a DCM study of the roving paradigm. Neuroimage 42, 936–944. 10.1016/j.neuroimage.2008.05.018 18602841 PMC2640481

[B15] Garrido M. I. Kilner J. M. Kiebel S. J. Friston K. J. (2009a). Dynamic causal modeling of the response to frequency deviants. J Neurophysiol. 101, 2620–2631. 10.1152/jn.90291.2008 19261714 PMC2681422

[B16] Garrido M. I. Kilner J. M. Stephan K. E. Friston K. J. (2009b). The mismatch negativity: A review of underlying mechanisms. Clin. Neurophysiol. 120, 453–463. 10.1016/j.clinph.2008.11.029 19181570 PMC2671031

[B17] Gramfort A. Papadopoulo T. Olivi E. Clerc M. (2010). OpenMEEG: opensource software for quasistatic bioelectromagnetics. Biomed. Eng. 9, 45. 10.1186/1475-925X-9-45 20819204 PMC2949879

[B18] Heinke W. Kenntner R. Gunter T. C. Sammler D. Olthoff D. Koelsch S. (2004). Sequential effects of increasing propofol sedation on frontal and temporal cortices as indexed by auditory event-related potentials. Anesthesiology 100, 617–625. 10.1097/00000542-200403000-00023 15108977

[B19] Hsiao F. J. Cheng C. H. Liao K. K. Lin Y. Y. (2010). Cortico-cortical phase synchrony in auditory mismatch processing. Biol. Psychol. 84, 336–345. 10.1016/j.biopsycho.2010.03.019 20380866

[B20] Joos K. Gilles A. Van de Heyning P. De Ridder D. Vanneste S. (2014). From sensation to percept: the neural signature of auditory event-related potentials. Neurosci. Biobehav. Rev. 42, 148–156. 10.1016/j.neubiorev.2014.02.009 24589492

[B21] Kamali A. Flanders A. E. Brody J. Hunter J. V. Hasan K. M. (2014). Tracing superior longitudinal fasciculus connectivity in the human brain using high resolution diffusion tensor tractography. Brain Struct. Funct. 219, 269–281. 10.1007/s00429-012-0498-y 23288254 PMC3633629

[B22] Karamzadeh N. Medvedev A. Azari A. Gandjbakhche A. Najafizadeh L. (2013). Capturing dynamic patterns of task-based functional connectivity with EEG. Neuroimage 66, 311–317. 10.1016/j.neuroimage.2012.10.032 23142654 PMC3609939

[B23] Kasai K. Nakagome K. Itoh K. Koshida I. Hata A. Iwanami A.. (1999). Multiple generators in the auditory automatic discrimination process in humans. Neuroreport 10, 2267–2271. 10.1097/00001756-199908020-00008 10439446

[B24] Kitzbichler M. G. Henson R. N. Smith M. L. Nathan P. J. Bullmore E. T. (2011). Cognitive Effort Drives Workspace Configuration of Human Brain Functional Networks. J. Neurosci. 31, 8259–8270. 10.1523/JNEUROSCI.0440-11.2011 21632947 PMC6622866

[B25] Koelsch S. Heinke W. Sammler D. Olthoff D. (2006). Auditory processing during deep propofol sedation and recovery from unconsciousness. Clin. Neurophysiol. 117, 1746–1759. 10.1016/j.clinph.2006.05.009 16807099

[B26] Ku S. W. Lee U. Noh G. J. Jun I. G. Mashour G. A. (2011). Preferential inhibition of frontal-to-parietal feedback connectivity is a neurophysiologic correlate of general anesthesia in surgical patients. PLoS ONE 6:e25155. 10.1371/journal.pone.0025155 21998638 PMC3187752

[B27] Lau T. M. Gwin J. T. McDowell K. G. Ferris D. P. (2012). Weighted phase lag index stability as an artifact resistant measure to detect cognitive EEG activity during locomotion. J. Neuroeng. Rehabil. 9:47. 10.1186/1743-0003-9-47 22828128 PMC3488562

[B28] Lee U. Kim S. Noh G. J. Choi B. M. Hwang E. Mashour G. A. (2009). The directionality and functional organization of frontoparietal connectivity during consciousness and anesthesia in humans. Conscious. Cogn. 18, 1069–1078. 10.1016/j.concog.2009.04.004 19443244

[B29] Lee U. Ku S. Noh G. Baek S. Choi B. Mashour G. A. (2013). Disruption of Frontal – Parietal Communication. Anesthesiology 118, 1264–1275. 10.1097/ALN.0b013e31829103f5 23695090 PMC4346246

[B30] Lin Y. Y. Hsiao F. J. Shih Y. H. Yiu C. H. Yen D. J. Kwan S. Y.. (2007). Plastic phase-locking and magnetic mismatch response to auditory deviants in temporal lobe epilepsy. Cereb. Cortex 17, 2516–2525. 10.1093/cercor/bhl157 17204819

[B31] Makris N. Kennedy D. N. McInerney S. Sorensen A. G. Wang R. Caviness V. S.. (2005). Segmentation of subcomponents within the superior longitudinal fascicle in humans: a quantitative, *in vivo*, DT-MRI study. Cereb. Cortex 15, 854–869. 10.1093/cercor/bhh186 15590909

[B32] Marco-Pallarés J. Grau C. Ruffini G. (2005). Combined ICA-LORETA analysis of mismatch negativity. Neuroimage 25, 471–477. 10.1016/j.neuroimage.2004.11.028 15784426

[B33] Molholm S. Martinez A. Ritter W. Javitt D. C. Foxe J. J. (2005). The neural circuitry of pre-attentive auditory change-detection: an fMRI study of pitch and duration mismatch negativity generators. Cereb. Cortex 15, 545–551. 10.1093/cercor/bhh155 15342438

[B34] Myles P. S. Leslie K. McNeil J. Forbes A. Chan M. T. (2004). Bispectral index monitoring to prevent awareness during anaesthesia: the B-Aware randomised controlled trial. Lancet 363, 1757–1763. 10.1016/S0140-6736(04)16300-9 15172773

[B35] Näätänen R. Alho K. (1997). Higher-order processes in auditory-change detection. Trends Cogn. Sci. 1, 44–45. 10.1016/S1364-6613(97)01013-9 21223853

[B36] Näätänen R. Tervaniemi M. Sussman E. Paavilainen P. Winkler I. (2001). “Primitive intelligence” in the auditory cortex. Trends Neurosci. 24, 283–288. 10.1016/S0166-2236(00)01790-2 11311381

[B37] Nicol R. M. Chapman S. C. Vértes P. E. Nathan P. J. Smith M. L. Shtyrov Y.. (2012). Fast reconfiguration of high-frequency brain networks in response to surprising changes in auditory input. J. Neurophysiol. 107, 1421–1430. 10.1152/jn.00817.2011 22170972 PMC3311691

[B38] Novitski N. Maess B. Tervaniemi M. (2006). Frequency specific impairment of automatic pitch change detection by fMRI acoustic noise: an MEG study. J. Neurosci. Methods 155, 149–159. 10.1016/j.jneumeth.2006.01.030 16530843

[B39] Opitz B. Rinne T. Mecklinger A. von Cramon D. Y. Schröger E. (2002). Differential contribution of frontal and temporal cortices to auditory change detection: fMRI and ERP results. Neuroimage 15, 167–174. 10.1006/nimg.2001.0970 11771985

[B40] Ozgoren M. Bayazit O. Kocaaslan S. Gokmen N. Oniz A. (2010). Brain function assessment in different conscious states. Nonlinear Biomed. Phys. 4(Suppl. 1):S6. 10.1186/1753-4631-4-S1-S6 20522267 PMC2880803

[B41] Paavilainen P. (2013). The mismatch-negativity (MMN) component of the auditory event-related potential to violations of abstract regularities: a review. Int. J. Psychophysiol. 88, 109–123. 10.1016/j.ijpsycho.2013.03.015 23542165

[B42] Ranlund S. Adams R. A. Díez Á. Constante M. Dutt A. Hall M. H.. (2016). Impaired prefrontal synaptic gain in people with psychosis and their relatives during the mismatch negativity. Hum. Brain Mapp. 37, 351–365. 10.1002/hbm.23035 26503033 PMC4843949

[B43] Rinne T. Alho K. Ilmoniemi R. J. Virtanen J. Näätänen R. (2000). Separate time behaviors of the temporal and frontal mismatch negativity sources. Neuroimage 12, 14–19. 10.1006/nimg.2000.0591 10875898

[B44] Rosburg T. (2003). Left hemispheric dipole locations of the neuromagnetic mismatch negativity to frequency, intensity and duration deviants. Cogn. Brain Res. 16, 83–90. 10.1016/S0926-6410(02)00222-7 12589892

[B45] Rosburg T. Trautner P. Dietl T. Korzyukov O. A. Boutros N. N. Schaller C.. (2005). Subdural recordings of the mismatch negativity (MMN) in patients with focal epilepsy. Brain 128, 819–828. 10.1093/brain/awh442 15728656

[B46] Schall U. Johnston P. Todd J. Ward P. B. Michie P. T. (2003). Functional neuroanatomy of auditory mismatch processing: an event-related fMRI study of duration-deviant oddballs. Neuroimage 20, 729–736. 10.1016/S1053-8119(03)00398-7 14568447

[B47] Schmahmann J. D. Smith E. E. Eichler F. S. Filley C. M. (2008). Cerebral white matter: Neuroanatomy, clinical neurology, and neurobehavioral correlates. Ann. N Y. Acad. Sci. 1142, 266–309. 10.1196/annals.1444.017 18990132 PMC3753195

[B48] Schmidt A. Diaconescu A. O. Kometer M. Friston K. J. Stephan K. E. Vollenweider F. X. (2013). Modeling ketamine effects on synaptic plasticity during the mismatch negativity. Cereb. Cortex 23, 2394–2406. 10.1093/cercor/bhs238 22875863 PMC3767962

[B49] Shestakova A. Brattico E. Huotilainen M. Galunov V. Soloviev A. Sams M.. (2002). Abstract phoneme representations in the left temporal cortex: magnetic mismatch negativity study. Neuroreport 13, 1813–1816. 10.1097/00001756-200210070-00025 12395130

[B50] Tadel F. Baillet S. Mosher J. C. Pantazis D. Leahy R. M. (2011). Brainstorm: a user-friendly application for MEG/EEG analysis. Comput. Intell. Neurosci. 2011:879716 10.1155/2011/879716 21584256 PMC3090754

[B51] Vinck M. Oostenveld R. Van Wingerden M. Battaglia F. Pennartz C. M. (2011). An improved index of phase-synchronization for electrophysiological data in the presence of volume-conduction, noise and sample-size bias. Neuroimage 55, 1548–1565. 10.1016/j.neuroimage.2011.01.055 21276857

[B52] Watts D. J. Strogatz S. H. (1998). Collective dynamics of “small-world” networks. Nature 393, 440–442. 9623998 10.1038/30918

[B53] Wehner D. T. Ahlfors S. P. Mody M. (2007). Effects of phonological contrast on auditory word discrimination in children with and without reading disability: a magnetoencephalography (MEG) study. Neuropsychologia 45, 3251–3262. 10.1016/j.neuropsychologia.2007.06.018 17675109 PMC2147041

